# National trends in the United States of America physician assistant workforce from 1980 to 2007

**DOI:** 10.1186/1478-4491-7-86

**Published:** 2009-11-26

**Authors:** Xiaoxing Z He, Ellen Cyran, Mark Salling

**Affiliations:** 1 Department of Health Sciences, Cleveland State University, 2121 Euclid Avenue HS 122, Cleveland, OH 44115, USA; 2 Northern Ohio Data & Information Service, Cleveland State University, 1717 Euclid Avenue, Cleveland, OH 44115, USA

## Abstract

**Background:**

The physician assistant (PA) profession is a nationally recognized medical profession in the United States of America (USA). However, relatively little is known regarding national trends of the PA workforce.

**Methods:**

We examined the 1980-2007 USA Census data to determine the demographic distribution of the PA workforce and PA-to-population relationships. Maps were developed to provide graphical display of the data. All analyses were adjusted for the complex census design and analytical weights provided by the Census Bureau.

**Results:**

In 1980 there were about 29 120 PAs, 64% of which were males. By contrast, in 2007 there were approximately 97 721 PAs with more than 66% of females. In 1980, Nevada had the highest estimated rate of 40 PAs per 100 000 persons, and North Dakota had the lowest rate (three). The corresponding rates in 2007 were about 85 in New Hampshire and ten in Mississippi. The levels of PA education have increased from less than 21% of PAs with four or more years of college in 1980, to more than 65% in 2007. While less than 17% of PAs were of minority groups in 1980, this figure rose to 23% in 2007. Although nearly 70% of PAs were younger than 35 years old in 1980, this percentage fell to 38% in 2007.

**Conclusion:**

The trends of sustained increase and geographic variation in the PA workforce were identified. Educational level, percentage of minority, and age of the PA workforce have increased over time. Major causes of the changes in the PA workforce include educational factors and federal legislation or state regulation.

## Background

The physician assistant (PA) profession of the United States of America (USA) emerged in the late 1960s, and has continued to thrive, becoming internationally recognized [1-3]. As health care professionals, PAs are licensed to practice medicine with physician supervision [4]. PAs' practices are not only in the areas of primary care, internal medicine, family medicine, pediatrics, obstetrics, and gynecology, but also in surgery and the surgical subspecialties. Physicians may delegate to PAs those medical duties that are within the physician's scope of practice and the PA's training and experience. Therefore, a broad range of diagnostic and therapeutic services are delivered by PAs to diverse populations in rural and urban settings. Because of the close working relationship between PAs and physicians, PAs are educated in a medical model designed to complement physician training [4]. The intensive PA education programs are accredited by the Accreditation Review Commission on Education for the Physician Assistant (ARC-PA). The average PA program runs approximately 26 months [4]. Graduation from an accredited PA program and passage of the national certifying program, developed by the National Commission on Certification of PAs (NCCPA), are required for state licensure. Federal or state laws and regulations affect PA workforce development and practice management [5]. The sustained growth of the PA workforce appears to be supported by federal Title VII of the U.S. Public Health Service Act, in response to skyrocketing medical expenditures, the physician shortage, and the primary care shortage crisis [6-11].

The physician shortage and the aging population make cost containment a critical issue [12-14]. A cost-effective way to meet the aging population's primary care needs is the PA model [15,16]. As the growth of the PA profession, it is important to understand the trends of changes in the PA workforce, in order to promote health education and disease prevention for improving the population's health [17-21]. Furthermore, evidence from public health system research indicates that the population's health is inevitably influenced by national policies and optimal supply of medical workforce [22]. However, there is not much literature regarding the current supply of the PA profession. While Larson et al. has attempted to describe the status of the PA workforce, the limitations are lack of current data and population information [23].

Using nationally-representative population data for 1980, 1990, 2000, 2005, and 2007, we examined the overall trends of changes in the PA workforce. As part of this analysis, we also examined the demographic characteristics and socioeconomic dimensions of the PA workforce, and PA-to-population relationships nationwide.

## Methods

### Sources of data

The sources of data were the 1980, 1990, and 2000 U.S. decennial Census and the 2005 and 2007 American Community Survey (ACS). For this analysis, the Integrated Public Use Microdata Sample (IPUMS) was used. The IPUMS data is the Public Use Microdata Sample (PUMS), released by the U.S. Census Bureau and enhanced for longitudinal research [24]. The IPUMS draws its sample in all 3141 counties (or county equivalents) in the USA [24-30]. The IPUMS data for the 1980, 1990, and 2000 are from the 'long form' samples of the U.S. decennial Census in those years. The IPUMS data for 2005 and 2007 are from the annual ACS. The ACS is a rolling sample through the year and is adjusted to the Census Bureau's independent population estimates program [24]. The ACS protocol calls for a sequential contact with a mixed-mode survey, resulting in a high (over 95%) response rate [24]. With the use of IPUMS data, the differences in the surveys' definitions of occupations over time are resolved.

### Study variables

In all of the IPUMS-USA data since 1980, respondents were asked to report their job activity and occupation [25,26]. Participants reported whether they worked at a private-for-profit; private not-for-profit; local, state, or federal government; were self-employed; or worked without pay in farm and family business. Participants also described the industry in which they worked, and responded to a variety of other employment questions, including their occupation. The PAs were identified in the 1980, 1990, 2000, 2005, 2007 IPUMS-USA data by the available code '106' for physicians' assistants, classified under the category of professional specialty occupations [27].

Over the 27 years, the only period of major change on the coding of occupation was between 1990 and 2000. Basically, the 1990 Census code '106' was matched directly to the 2000 Census code '311' for physicians' assistants [28]. The 1990 Census code '106' was equivalent to 2000 Census code '311', plus the code '340' for emergency medical technicians (EMT) and paramedics, and the code '365' for medical assistants and other health care support occupations. The 2000 Census code '311' would be equivalent to the 1990 Census code '106' and 5% of the code '208' for health technologists and technicians. However, the standard job title of 'physicians' assistants' remained the same as a single occupation over time. The change of code definition from '106' to '311' was based on keeping the number in that occupation, and earnings, consistent.

The occupation code/definition change might account for some but not all demographic changes between 1990 and 2000. Nevertheless, it does not account for any changes between 1980-1990 or 2000-2005, and 2007. The consistent category system for 1960-2000 Census occupations was described in the Bureau of Labor Statistics (BLS) working paper: "we analyze employment levels, average earnings levels, and earnings variance in our occupation categories over time, compare these to similar trends for occupations defined in the occ1950IPUMS classification, and test both classifications for consistency over time" [28]. Thus, we were able to analyze the characteristics of such occupations as physician and PA. We analyzed these study variables with a focus on the PA profession to describe the trends of the PA workforce. This is the first step of a serial analysis (forthcoming) to examine the changes in healthcare workforce structure in order to identify the impact on health services utilization or medical expenditures, and to project the optimal supply of the nation's medical workforce.

### Analysis

We applied the Geographical Information System (GIS) analysis to examine the patterns of changes in the PA workforce from 1980 to 2007. Maps were developed to provide an intuitive graphical display of the data. The analysis documented how demographic trends and the geographic distribution of the PA workforce have changed over time, with a focus on the most recent period from 2000 to 2007. In addition to analyzing overall trends, we assessed the degree of variation in the PA workforce distribution across the states. Furthermore, we examined the ratio of PAs to population by state. The analysis was supplemented with data on the PA profession's average hourly and annual wages from the Occupational Employment Statistics (OES) from the U.S. Department of Labor. Appropriate statistical tests have been applied, especially to the 2005 and 2007 Census data, given their relatively small sample size (1% sample), to ensure the estimates are reliable. All analyses were adjusted for the complex census design and analytical weights provided by the Census Bureau.

## Results

### Overall trends of the PA workforce

The estimated numbers of PAs more than tripled from 1980 to 2007. In 1980, nearly 64 per cent of PAs were male. By 2007, more than 66 per cent of PAs were female (Table 1). From 1980 to 1990, there was a decrease in the number of PAs. Although there was only a slight increase of male PAs, it indicated more than threefold increase of female PAs from 1990 to 2000. In the five-year period between 2000 and 2005, there was an increase of more than 10 000 PAs among both males and females. In the years of 2005 to 2007, there was a small increase of male PAs (about twelve hundred), and sustained growth of female PAs (over fourteen thousand).

**Table 1:** Estimated employed PAs by gender and education in the USA, 1980-2007

Gender & Education	1980	1990	2000	2005	2007
**Total: N**	**29 120**	**23 618**	**56 922**	**82 135**	**97 721**

**Male: N (%)**					

**<12^th ^grade**	1520 (5.2)	375 (1.6)	266 (0.5)	317 (0.4)	413 (0.4)

**12^th ^grade**	4900 (16.8)	1332 (5.6)	1170 (2.1)	1894 (2.3)	1044 (1.1)

**1-3 years of college**	7580 (26.0)	6365 (26.9)	4831 (8.5)	5402 (6.6)	6535 (6.7)

**4+ years of college**	4500 (15.5)	4270 (18.1)	14 718 (25.9)	23 504 (28.6)	24 384 (24.9)

**Total**	18 500 (63.5)	12 342 (52.2)	20 985 (37.0)	31 117 (37.9)	32 376 (33.1)

**Female: N (%)**					

**<12^th ^grade**	1400 (4.8)	395 (1.7)	447 (0.8)	574 (0.7)	1381 (1.4)

**12^th ^grade**	5340 (18.3)	2478 (10.5)	4066 (7.1)	5119 (6.2)	5404 (5.5)

**1-3 years of college**	2340 (8.0)	5259 (22.3)	12 423 (21.8)	14 835 (18.1)	19 100 (19.5)

**4+ years of college**	1540 (5.3)	3144 (13.3)	19 001 (33.4)	30 490 (37.1)	39 460 (40.4)

**Total**	10 620 (36.4)	11 276 (47.8)	35 937 (63.1)	51 018 (62.1)	65 345 (66.8)

* Estimates are adjusted using weights provided by the Census Bureau. † While 95% Confidence Intervals are not listed due to space limitations, the estimates with appropriate statistical testing conducted on the various differences are reliable according to the Census Bureau. ‡The added percentage may not be 100, due to rounding.

### Demographic characteristics of the PA workforce

The educational background of PAs has improved from less than 21 per cent of PAs with four or more years of college in 1980, to more than 65 per cent in 2007. In 1980, nearly 5 per cent of the PAs had less than a twelfth grade education. By 2007, only 1 per cent of the PAs had an education background of less than twelfth grade. The increase in educational attainment in the PA profession is especially notable for females (Table 1). In 1980, about 5 per cent of female PAs had four or more years of college. Dramatically, over 40 per cent of female PAs had four or more years of college by 2007.

In terms of racial and ethnic profile, while fewer than 17 per cent of PAs were minority races (non-White) in 1980, the estimated percentage of PAs that were minorities increased to 23 per cent by 2007 (Table 2). Asian American PAs had the greatest percentage increase over time. Between 1980 and 2007, Asian American PAs increased threefold - growing from two to six per cent of all PAs.

**Table 2:** Estimated employed PAs by age and race/ethnicity in the USA, 1980-2007

Age & ace/ethnicity, N (%)	1980	1990	2000	2005	2007
**<35**	20 240 (69.5)	13 662 (57.8)	21 990 (38.6)	30 218 (36.8)	36 923(37.8)

**35-44**	5020 (17.2)	6985 (29.6)	17 663 (31.0)	23 205 (28.3)	29 302(29.9)

**45-54**	2160 (7.4)	2028 (8.6)	13 118 (23.0)	20 326 (24.7)	20 347(20.8)

**55-64**	980 (3.4)	823 (3.5)	3360 (5.9)	7222 (8.8)	9761 (9.9)

**65-74**	520 (1.8)	58 (0.2)	618 (1.1)	927 (1.1)	1064 (1.1)

**75+**	200 (0.7)	62 (0.3)	173 (0.3)	237 (0.3)	324 (0.3)

**White NH**	24 160 (82.9)	18 921 (80.1)	43 628 (76.6)	60 962 (74.2)	75 408 (77.2)

**Black NH**	2780 (9.5)	2053 (8.7)	4830 (8.5)	7707 (9.4)	7606 (7.8)

**American Indian/Native NH**	120 (0.4)	183 (0.8)	390 (0.7)	481 (0.6)	470 (0.5)

**Asian NH**	480 (1.6)	1118 (4.7)	2457 (4.3)	4087 (4.9)	5382 (5.5)

**Native Hawaiian NH**	N/A	45 (0.2)	78 (0.1)	N/A	N/A

**Some other races NH**	N/A	N/A	54 (0.1)	147 (0.2)	198 (0.2)

**2+ major race groups NH**	N/A	N/A	1028 (1.8)	437 (0.5)	604 (0.6)

**Hispanic or Latino**	1580 (5.4)	1298 (5.5)	4457 (7.8)	8314 (10.1)	8053 (8.2)

* Estimates are adjusted using weights provided by the Census Bureau. † While 95% Confidence Intervals are not listed due to space limitations, the estimates with appropriate statistical testing conducted on the various differences are reliable according to the Census Bureau. ‡ NH: Not Hispanic

The age profile of the PA workforce had also undergone significant change. While nearly 70 per cent of PAs were less than 35 years old in 1980, this estimated percentage fell to 38 per cent in 2007 (Table 2). The most remarkable changes occurred among the 45 to 54 age cohort. In 1980, this age group composed of only seven per cent of the PA workforce; by 2007, more than 20 per cent were 45 to 54 years old. Other noticeable changes were among the 35 to 44 and 55 to 64 years old cohorts. In 1980, an estimated 17 per cent of the PAs were 35 to 44 years old. By 2007 the estimated percentage had increased to about 30 per cent - nearly doubling its share of the PA workforce in 27 years. While only three per cent of the PAs were 55 to 64 years old in 1980, almost 10 per cent of all PAs were estimated to be in that age group by 2007.

### PA-to-population ratios and wages

Ratios of PAs per 100 000 persons varied greatly among the states for all years in the study (Table 3). In 1980, Nevada had the highest estimated ratio - 40 PAs per 100 000 persons, followed by Florida (29.8), and Alabama (26.2). North Dakota had the lowest ratio - three PAs per 100 000 persons. Other states with low ratios in 1980 included Vermont (3.9), and Wyoming (4.3). In 2007, the highest ratio of PAs per 100 000 persons were 84.7 in New Hampshire, 75.3 in Maine, and 63.0 in Rhode Island. The three states with the lowest ratios were Mississippi (10.4), New Mexico (11.4), and Missouri (11.7).

**Table 3:** Estimated rates of PAs per 100 000 persons and wages by states in the USA, 1980-2007

States	1980	1990	2000	2005	2007	2007 Hourly mean wages	2007 Annual mean wages
Alabama	26.2	12.1	24.6	13.8	39.9	33.04	68 720

Alaska	14.9	19.5	25.0	16.9	54.3	43.01	89 460

Arizona	23.5	9.5	25.9	42.4	37.5	37.35	77 690

Arkansas	5.2	7.7	8.4	18.8	21.3	31.97	66 490

California	15.6	9.4	20.1	25.8	31.4	37.56	78 120

Colorado	21.5	14.9	27.9	34.1	31.3	36.56	76 050

Connecticut	7.1	14.8	34.5	73.0	38.6	43.76	91 010

Delaware	16.8	5.9	24.0	9.7	57.3	38.8	80 710

DC	12.5	9.1	28.0	58.1	49.8	36.96	76 880

Florida	29.8	10.8	29.8	45.3	35.4	39.23	81 600

Georgia	15.4	15.7	26.2	34.1	60.7	37.58	78 170

Hawaii	20.7	27.0	12.1	6.9	46.5	30.79	64 040

Idaho	14.8	10.7	30.5	12.4	21.9	30.15	62 700

Illinois	18.9	9.6	17.5	23.0	21.3	33.02	68 680

Indiana	10.6	12.7	15.9	19.1	22.6	32.78	68 190

Iowa	10.3	8.4	24.3	35.2	61.1	36.6	76 130

Kansas	19.5	13.6	26.5	45.1	58.7	38.06	79 170

Kentucky	9.8	4.5	24.8	36.2	27.5	36.13	75 160

Louisiana	20.4	9.7	18.5	37.3	31.3	27.24	56 650

Maine	10.7	18.1	59.3	39.1	75.3	39.88	82 960

Maryland	19.0	15.7	24.6	45.3	56.9	39.99	83 190

Massachusetts	6.6	9.3	29.2	19.9	45.6	39.29	81 720

Michigan	14.5	9.3	22.9	38.2	26.5	38.1	79 240

Minnesota	7.9	11.7	24.3	45.4	40.3	40.04	83 280

Mississippi	7.9	8.0	19.4	36.2	10.4	20.27	42 160

Missouri	21.2	13.4	16.0	15.9	11.7	29.44	61 240

Montana	12.7	3.3	25.2	32.1	20.8	30.98	64 440

Nebraska	12.7	15.6	23.3	36.1	58.5	37.98	79 010

Nevada	40.0	11.0	18.3	11.7	17.4	40.3	83 820

New Hampshire	6.5	4.6	36.8	19.4	84.7	38.91	80 920

New Jersey	11.4	9.7	13.8	27.5	25.3	42.69	88 800

New Mexico	16.9	14.1	27.4	35.6	11.4	24.19	50 320

New York	13.8	12.1	31.2	43.6	48.6	39.98	83 160

North Carolina	14.3	12.8	33.3	36.4	42.0	37.87	78 760

North Dakota	3.1	12.5	28.3	55.3	39.3	33.69	70 080

Ohio	11.7	11.7	22.9	21.3	34.3	38.12	79 280

Oklahoma	14.5	4.0	27.9	16.9	29.8	38.75	80 600

Oregon	12.2	4.7	22.7	33.5	33.9	39.16	81 460

Pennsylvania	13.8	11.3	25.7	26.2	48.7	32.39	67 370

Rhode Island	6.3	N/A	22.3	39.8	63.0	36.73	76 400

South Carolina	17.3	11.8	17.6	27.4	25.1	35.31	73 450

South Dakota	11.6	16.7	13.2	99.6	21.4	37.46	77 920

Tennessee	15.7	12.7	23.6	48.2	29.8	35.38	73 590

Texas	13.9	14.6	21.5	34.8	32.0	39.4	81 960

Utah	16.4	8.1	28.3	36.9	31.8	41.52	86 360

Vermont	3.9	26.7	19.2	13.4	35.9	39.11	81 340

Virginia	13.8	4.9	20.5	17.4	38.7	30.46	63 350

Washington	16.5	12.9	29.8	35.1	40.4	41.45	86 210

West Virginia	17.4	19.8	27.5	50.5	30.7	36.03	74 950

Wisconsin	14.0	9.5	28.8	32.9	42.0	38.53	80 140

Wyoming	4.3	N/A	7.5	N/A	51.8	31.29	65 080

* Estimates are adjusted using weights provided by the Census Bureau. † While 95% Confidence Intervals are not listed due to space limitations, the estimates with appropriate statistical testing conducted on the various differences are reliable according to the Census Bureau. ‡ DC: District of Columbia.

Data on salaries in 2007 showed that Connecticut's PAs earned the highest hourly mean wages ($43.8) and annual mean wages ($91 010). The lowest hourly mean wages were $20.3 in Mississippi, and it also had the lowest annual mean wages at $42 160 (Table 3).

### Geographic shifts in the PA workforce

In 1980, the top five states with the highest estimated numbers of PAs were California (3120), Florida (2520), New York (1920), Illinois (1800), and Texas (1740). Conversely, the five states with the lowest estimated number of PAs were North Dakota (20), Vermont (20), Wyoming (20), New Hampshire (40), and Alaska (40). The geographic distribution of the PA workforce has been changing over time. By 2007, New York employed the greatest estimated number of PAs (9010), closely followed by California (9004), Texas (6646), Pennsylvania (5874), and Florida (5806). North Dakota had the lowest number of PAs (106) employed in 2007. Two other states that employed fewer than 200 PAs in 2007 were South Dakota (170) and Montana (199) (data not shown).

**Figure 1:**
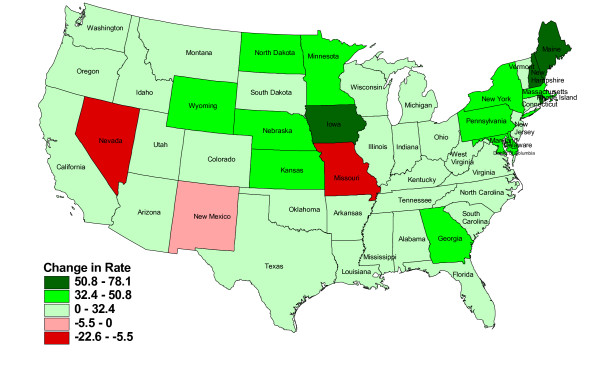
**Change in the estimated rate of PAs per 100 000 persons, USA, 1980-2007**. Prepared in January 2009 by Northern Ohio Data & Information Service, NODIS, The Urban Center, Maxine Goodman Levin College of Urban Affairs, Cleveland State University, January 2009.

**Figure 2:**
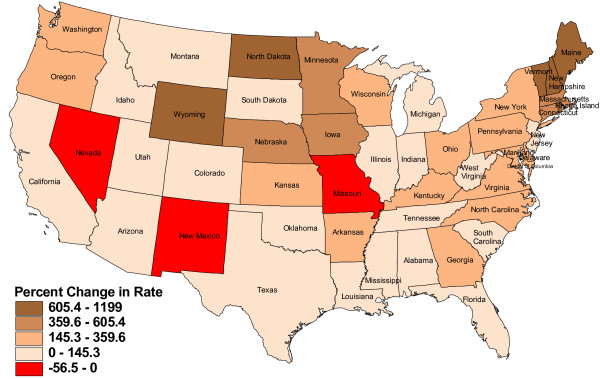
**Percent change in the estimated rate of PAs per 100 000 persons, 1980-2007**. Prepared in January 2009 by Northern Ohio Data & Information Service, NODIS, The Urban Center, Maxine Goodman Levin College of Urban Affairs, Cleveland State University, January 2009.

Figure 1 and Figure 2 display the absolute changes and the percentage changes in the rates of PAs per 100 000 persons across the states. The ratios of PAs to population had increased since 1980 in all but three states - Missouri, Nevada, and New Mexico. The greatest growth was in New England and upper Midwest states. Maine, New Hampshire, and Iowa had the greatest positive changes in the rates of PAs per 100 000 persons (Figure 1). The states with the largest percentage increase in the rate of PAs to population were Maine, Vermont, New Hampshire, North Dakota, and Wyoming (Figure 2).

## Discussions

In this study, we sought to identify the trends of the PA workforce from 1980 to 2007, based on the estimates from the USA Census Bureau. A major trend is the increase in PA workers, with the greatest expansion of PA workforce between 2000 and 2005. In addition, levels of education, percentage of minority, and age of the PA workforce have increased. One notable change in PA workforce is the ratio of males to females, from about 1.7 in 1980 down to 0.5 by 2007. Another remarkable change is that the rates of PAs to population and the average wages of PAs vary greatly across the 50 states and District of Columbia. Furthermore, there is a growing concentration of the PA profession in New England and upper Midwest states over the 27 years of study period.

The greatest expansion of PA workforce in 2000 to 2005 likely resulted from the third period of the federal Title VII Public Health Service Act which supported training of health professions in medicine and dentistry [6-8]. The first period, from 1963 to 1975, appeared to lead the emergence of the PA profession. Title VII support in the second period, from 1976 to 1991, seemingly marked the establishment of primary care disciplines and related divisions in all medical schools [8]. Meanwhile, there was a small decrease in male PAs and a slight increase in female PAs, as shown in our findings. In the third era, from 1992 to present, national policy goals have emphasized caring for vulnerable populations, greater diversity in the health professions, and innovative curricula to prepare trainees [8]. Apparently, the third period of Title VII support induced a sustained growth of PA workforce, especially the expansion between 2000 and 2005. The findings of increased percentage of minority PAs and levels of PA education in this study could serve as direct evidence of the targeted outcomes of the Title VII third era's national policy goals. The correlation between the federal Title VII Public Health Service Act and the PA workforce expansion could be empirically tested by the planned follow-up analysis.

While we see favorable increases in the total numbers of PAs, the levels of education, and the percentage of minority PAs, an alarming sign is also indicated in our study. Although it is still a relatively young medical workforce, the PA profession is growing older - a reflection of similar trends in other professions and in the nation's population in general. To keep up with the PA profession's original goals of meeting the aging population's primary care needs, it is imperative to develop innovative recruitment strategies for PA programs to enroll new PA students in their 20s and early 30s. This is critically important in building a sustained supply of the PA workforce.

Recruiting younger PA students might also help to balance the ratio of males to females, since the 'feminization' of the PA profession appears to be the consequences of more education, observed in females [31]. In addition, a previous study suggests that younger PA students are more likely to stay and practice in rural areas if they are recruited and receive training there [32]. Therefore, recruiting younger PA students locally would help to meet the original Title VII goals of filling the existing gap of the physician shortage and enhancing the primary care practice in rural or underserved areas.

Our findings have shown a large variation among the 50 states and District of Columbia with regard to the rates of PAs per 100 000 persons and the PAs' average wages. Some possible explanations include the changes over time in state laws for PA practice regulations, the delegation of services agreements (DSA), and the numbers of PA educational programs. The American Academy of Physician Assistants (AAPA) website has the detailed summaries of state laws and regulations [5]. A comparative reading of the summary clauses of state regulations indicates that a favorable practice environment, in particular the flexibility of physician supervision requirements [5], appears to be the most important factor in encouraging the growth of the PA workforce. For example, New Hampshire, Maine, and Rhode Island - the three states with the highest rates of PAs per 100 000 persons in 2007, had relatively flexible supervision requirements. In these three states, a physician was not required to be physically present, as long as the physician was easily contactable to advise the PA through easy-to-use and effective electronics or telecommunications.

However, more restricted supervision requirements existed for the three states with the lowest rates of PAs per 100 000 persons in 2007. Mississippi requires on-site presence of a physician for the first 120 days of care, and a supervising physician must review and initial 10 per cent of the PA-written charts monthly. New Mexico demands immediate communication between the physician and the PA to specify what services may be provided. Missouri mandates that the attending physician must practice in the same facility as the PA, and be present at least 66 per cent of the time when a PA is providing care.

Furthermore, the enacted dates that PAs were licensed, registered, or certified to practice had inevitable impact on the variations of PAs' ratios per 100 000 persons and PAs' average wages. In 2000, Mississippi--the state with the lowest rate of PAs per 100 000 persons and the lowest average wages in 2007--was the last state to establish the statute for PA practice [5]. Our study suggests the necessity for the federal government to standardize PA practice regulations across the nation in order to effectively allocate workforce, improve quality of care, and reduce health disparities.

Moreover, we posit that the availability or the numbers of PA educational programs played a chief role in influencing the geographic distribution of the PA workforce. Based on a list of all accredited PA educational programs by the AAPA [4], of the three states with the lowest rates of PAs per 100 000 persons in 1980, two states (Vermont and Wyoming) did not have any PA educational programs. Similarly, no PA educational programs were found among two of the three states in 2007 with the lowest ratios of PAs to population (Mississippi and Missouri). Therefore, a national approach or coordinated strategy for training and retaining PAs is recommended in order to sustain the PA workforce supply and balance the distribution of the PA workforce more equitably.

Limitations associated with the data should be noted. Like all surveys, the USA Census surveys are subject to potential problems of sampling error and response bias. The PA samples are relatively small for some states in 1980. Data on their attributes at the national level are more reliable and the relatively high response rates minimize the potential for selection bias. In addition, the measures of occupation and job activities were self-reported, and might contribute to reporting bias. Finally, the estimated numbers of employed PAs appear to be higher than those estimates of clinically active PAs in the AAPA survey report. The differences in the estimates can be attributed to the different assumptions or survey sampling methods and questionnaires used for data collection. Among the study's strengths are innovative analysis ideas and unique research designs to explore a topic without much existing literature.

As a first step in identifying the optimal structure of the nation's medical workforce, our study informs the USA policy by providing new information about national trends in the PA workforce from 1980 to 2007. Further studies are necessary to inform the development of national policies with regard to the cost-effectiveness of various supply patterns for meeting primary care needs, especially in rural or underserved areas, and the impact of various supply patterns on medical expenditures in the nation's health care system.

## Competing interests

The authors declare that they have no competing interests.

## Authors' contributions

XH conceived and designed the study, interpreted the preliminary results, and was responsible for writing the paper. EC completed preliminary analyses. MS made geographic maps and helped to edit the draft. All authors read and approved the final manuscript.
